# Primary Sjögren's syndrome with diffuse cystic lung changes developed systemic lupus erythematosus: a case report and literature review

**DOI:** 10.18632/oncotarget.16010

**Published:** 2017-03-08

**Authors:** Xiao Liu, Hao Li, Yunhong Yin, Dedong Ma, Yiqing Qu

**Affiliations:** ^1^ Department of Respiratory Medicine, Qilu Hospital of Shandong University, Jinan, China

**Keywords:** Sjögren's syndrome, systemic lupus erythematosus, diffuse cystic lung changes

## Abstract

Sjögren's syndrome (SS) is a chronic inflammatory autoimmune disease that can occur as a unique existence (primary Sjögren's syndrome) or merge with other systemic diseases like systemic lupus erythematosus (SLE), rheumatoid arthritis or systemic sclerosis (secondary Sjögren's syndrome). Data on the two diseases occurrence order are inadequate. Primary Sjögren's syndrome (pSS) may relatively uncommonly lead to diffuse cystic lung changes. We represent a female who was diagnosed pSS with diffuse cystic lung alterations developed SLE two years later. SS was diagnosed on account of the existence of dryness of eye and mouth, Schirmer's test, biopsy of the minor salivary glands of her lip, positive anti-SSA and anti-SSB antibody in the serum. Chest computed tomography image showed bilateral diffuse cystic changes with a wide variation in cyst size and distribution. SLE was finally diagnosed based on bilateral lower limb skin rash, gonarthritis and omarthritis, low level of complement, antinuclear antibody 1:640 and positive antibodies to double-stranded DNA. Improvement was achieved with therapy of corticosteroids, hydroxychloroquine and antibiotics. This report provides us clinical, diagnosis and treatment perception of SS-onset SLE as patient presenting diffuse cystic lung changes.

## INTRODUCTION

Sjögren's syndrome (SS) is a chronic systemic disease characterized by lymphocytic infiltration of exocrine glands, it can occur as a distinct entity (primary Sjögren's syndrome) or in association with other autoimmune diseases like systemic lupus erythematosus (SLE) (secondary Sjögren's syndrome). When SS is concurrent with SLE, the former is commonly considered as secondary to the later although records on which to base this viewpoint are inadequate [1–7]. Some studies have shown that primary Sjögren's syndrome (pSS) with extraglandular manifestations can precede SLE by several years [5, 8–17].

Pulmonary disease in SS has a extensive rate of 9% to 90% in different case series [18–21]. Pulmonary cysts have been reported in several studies to occur in 7.4% to 46.2% of patients with SS [18–20, 22–24]. Few studies have evaluated the nature of cystic abnormalities on chest imaging in patients with SS [18,19, 22, 25, 26]. Typical characteristics of the cystic pattern associated with SS on high resolution computed tomography (HRCT) imaging include a broad differentiation in cyst size and inner construction within cysts, positional simplification of parenchymal structure, perivascular and generally basilar dominating allocation and frequent association with ground glass opacities and nodules [26].

We show here a female patient who was diagnosed pSS with diffuse cystic lung changes developed SLE two years later. This report could be useful for coming research of diagnosis and treatment for this type of disease.

## CASE PRESENTATION

**Figure 1:**
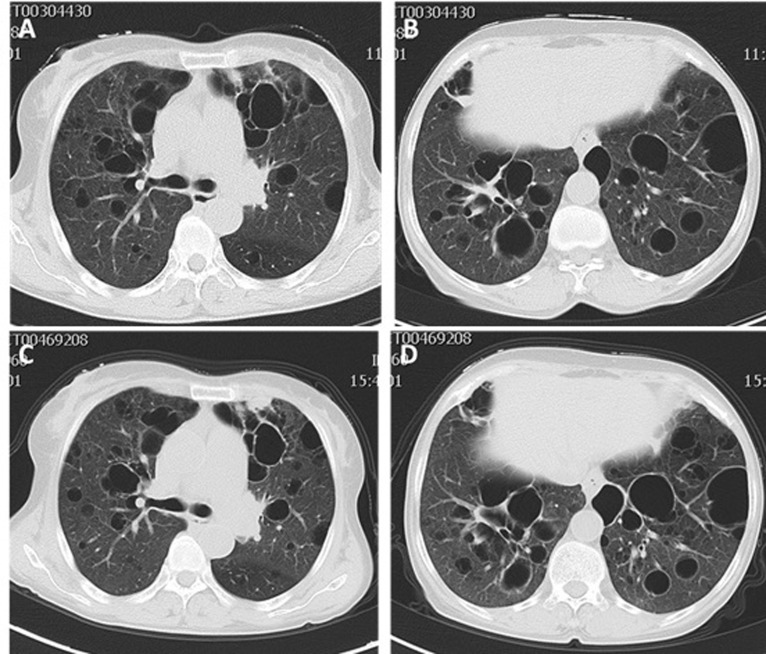
**A**.-**B**. Chest high resolution computed tomography (HRCT) images in 2014 showing bilateral diffuse thin walled cystic changes with a wide variation in cyst size and distribution. **C**.-**D**. Chest HRCT images in 2016 which were almost unchanged from 2014.

A 56-year-old woman was hospitalized in Qilu Hospital of Shandong University in October 2014 because of a history of xerostomia, xerophthalmia, dry systemic skin for six years and intermittent cough, little white sputum, worsening dyspnea for three years. Physical examination revealed vesicular breath sounds in bilateral lungs. The remainder of physical examination was normal. Her laboratory work showed white blood cell (WBC) count 2100/mm^3^ (normal 4000-10000/mm^3^), neutrophil (NEU) count 1410/mm^3^ (normal 1800-6300/mm^3^), lymphocyte count 610/mm^3^ (normal 1100-3200/mm^3^), monocyte count 90/mm^3^ (normal 100-600/mm^3^), erythrocyte count 3.44×10^12^/L (normal 3.8-5.1×10^12^/L), erythrocyte sedimentation rate (ESR) 72mm/h (normal 0-18mm/h). Rheumatism laboratory examination showed anti-nuclear antibody (ANA) 1:640 positive (normal < 1:80), positive anti-SSA antibody and anti-SSB antibody (normal negative), quantitative anti-bodies to double-stranded DNA (dsDNA) 49.55 IU/ml (normal < 100IU/ml). Humoral immune test showed IgG 35 g/L (normal 7-16g/L), IgA 9.77 g/L (normal 0.7-4 g/L), C3 (complement component 3) 0.84 g/L (normal 0.9-1.8g/L), C4 (complement component 4) 0.093 g/L (normal 0.1-0.4g/L) (Table 1). Schirmer's test showed right eye 2mm/5min and left eye 5mm/5min (normal 10-45mm/5min). Chest computed tomography (CT) showed bilateral diffuse cystic changes with a wide variation in cyst size (Figure 1A-1B). The diameter length of vesicles ranged approximately from 3.2mm to 50.2mm. Cysts were thin walled and asymmetrically distributed throughout the lung. She was diagnosed pSS with diffuse cystic lung changes. After 8 days therapy of intravenous methylprednisolone (40mg qd), hydroxychloroquine (200mg bid) and antibiotics of cefuroxime (1.5g q12h), she felt xerostomia, xerophthalmia and respiratory symptoms relieved. The blood routine indexes also showed improvement from WBC count 2100/mm^3^ to 3640/mm^3^ and from NEU count 1410/mm^3^ to 2399/mm^3^. She was discharged home and had been taking prednisone (50mg qd) with hydroxychloroquine (200mg bid). She gradually reduced dosage of prednisone to zero, and stopped taking all drugs on Feb 2016.

**Table 1:** Laboratory values of serum

Variable of Serum	First admission(2014)	Second admission(2016)	Normal range in our unit
Anti-nuclear antibody	1:640	1:640	<1:80
Anti-SSA antibody	positive	positive	negative
Anti-SSB antibody	positive	positive	negative
Antibodies to dsDNA	49.55	290.51	<100 IU/ml
White blood cell	2100	4500	(4000, 10000)/mm^3^
Lymphocyte	610	1650	(1100, 3200)/mm^3^
C3	0.84	0.63	(0.9, 1.8)g/L
C4	0.093	0.108	(0.1, 0.4)g/L
IgG	35	21	(7, 16)g/L
IgA	9.77	2.94	(7, 4)g/L

(Normal range in our unit: normal range of clinical laboratory in Qilu Hospital of Shandong University)

Then she was admitted to our hospital again in March 2016 because of the aggravation of cough, dyspnea, xerostomia and xerophthalmia after drug withdrawal. She also reported accompanying clinical symptoms of painful shoulder-joints and knee-joints in the past month. She denied fevers, chills or shivers. She did not have any surgical history. Negative family history for coronary artery disease and ovarian cancer was confirmed. She had never smoked, used alcohol or illicit drugs and had no environmental exposures. Vital signs on admission showed heart rate of 84/minute, respiration rate of 21/minute, blood pressure of 128/78mmHg, axillary temperature 36.6°C. Physical examination revealed coarse breath sounds bilaterally. Her eyes and mouth were extremely dry. Sheet red rash was seen on bilateral lower limb skin. Swollen knee-joints were seen.

Laboratory examinations showed ANA 1:640 (normal < 1:80), dsDNA 290.51 IU/ml (normal < 100IU/ml), positive anti-SSA and anti-SSB antibody. Serologic humoral immunity showed IgG 21 g/L (normal 7-16g/L), IgA 2.94 g/L (normal 0.7-4 g/L), C3 0.63 g/L (normal 0.9-1.8g/L), and C4 0.108 g/L (normal 0.1-0.4g/L) (Table 1). Serum angiotensin-converting enzyme (SACE) was 12.40 U/L (normal 5-68U/L). Indices of urine routine test, liver function test, renal function test and tumor biomarkers were all in normal range.

**Figure 2:**
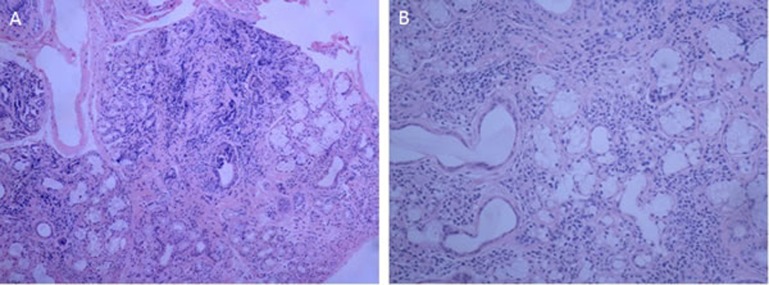
Histology of the little salivary glands in the lower lip revealing infiltration of lymphocytes in the glands **A**. (original magnification, ×100), **B**. (original magnification, ×200).

CT showed asymmetrically diffuse cystic changes with a wide variation in cyst size and distribution, which was almost unchanged from 2014 (Figure 1C-1D). Schirmer's test of her eyes revealed both eyes 0 mm/5min. Secretions of saliva were reduced. Biopsy of lower lip revealed chronic inflammation and lymphocytes infiltration of minor salivary glands (Figure 2). Pulmonary function test showed total lung capacity (TLC) 4.51L (predicted value 4.64L), vital capacity (VC) MAX 2.32L (predicted value 2.67L), forced vital capacity (FVC) 1.96L (predicted value 2.58L), forced expiratory volume in one second (FEV_1_) 1.34L (predicted value 2.17L), FEV_1_/predicted FEV_1_ value (FEV_1_%) 61.9%, FEV_1_/forced capacity ratio (FEV_1_/FVC) 68.31%, and diffusion function test showed carbon monoxide transfer factor in single breath (TLCO SB) 7.22 ml/min/mmHg (predicted value 21.78 ml/min/mmHg).

Eventually, this patient was diagnosed pSS presenting diffuse cystic lung changes developed SLE. During this hospitalization, she was started on oral hydroxychloroquine (200mg bid), intravenous methylprednisolone (80mg qd) and antibiotics of sulbactam and cefoperazone (2g q12h) therapy. On the seventh day of hospitalization, pulse therapy was discontinued and she was started on oral prednisone 60 mg daily with hydroxychloroquine (200 mg bid). She felt clinical symptoms improved again and laboratory examination showed ESR decreased from 69mm/h to 18mm/h. On the tenth day of hospitalization, the dosage of prednisone was reduced to 40 mg and she was discharged home.

In this case reported here, differential diagnoses like lymphangioleiomyomatosis (LAM) were unlikely. SACE was in normal range. Besides, she did not have medical history of pneumothorax.

## FOLLOW UP

The patient has been followed up for 9 months. She denied any aggravation of clinical symptoms. Hence no further imaging examinations or laboratory tests were pursued and she continued to be managed clinically.

## DISCUSSION

SS is a chronic systemic inflammatory disease with lymphocytic infiltration of exocrine glands. SS can exist as a unique presence (pSS) or in combination with other systemic diseases (sSS). While salivary and lacrimal glands are most ordinarily involved, impaired mucosal defense and glandular dysfunction inside of the lung may make it more vulnerable to pulmonary inflammation [20]. The case reported here was diagnosed Sjögren's syndrome in 2014 according to the American and European Consensus Group classification criteria (AECG-criteria) [27], and then developed SLE in 2016 based on the 2012 revision classification criteria for SLE [28]. HRCT images in 2014 and 2016 showed asymmetric multiple cysts of varying size and distribution in both lungs, and did not present an obvious progression in the size, number and distribution of cysts.

That SS can be coexistence with SLE was first reported in 1959 [29] and has since then been documented by several studies [1, 3–17, 30–41]. Reports indicated that 3.7%-19.6% SLE patients were accompanying SS [2, 4, 34–36]. However, rare published reports identified time sequence that pSS preceded SLE [5, 8–17] or SLE preceded SS [4–7] (Table 2). We generally comprehend specific clinical, laboratory, treatment and prognosis features that distinguish SLE-onset SS and SS-onset SLE from SS or SLE only patients. SLE-onset SS were older at SLE diagnosis, showed lower mortality and improved survival estimates when compared with SLE-only patients [4, 34]. SS-onset SLE were significantly older, showed a higher frequency of Raynaud's phenomenon, anti-SSA and anti-SSB, a lower frequency of severe renal involvement and lower mortality, and presented comparatively stable disease course and benign prognosis that required less vigorous treatment with glucocorticoids and/or immunosuppressant when compared with SLE-only patients [5, 11, 12]. SS-onset SLE were younger and had an increased frequency of arthritis, xerostomia, anticardiolipin antibodies and low levels of C3 and C4 in the serum, perivascular infiltrates in the salivary glands but a lower frequency of interstitial lung disease when compared with SS-only [11, 13].

**Table 2:** Summary of SS-SLE articles that identified time sequence of SS and SLE

Reference	Brief
Nossent et al. [4]	27 patients developed SS after a mean period of 48 months of 138 SLE patients.
BAER et al. [5]	In a group of 259 SS-SLE patients, SS preceded SLE by more than 1 year in 32 patients and SLE preceded SS in 207 patients.
Hernández-Molina et al. [6]	19 SLE-onset SS were diagnosed in a prospective cohort of 103 SLE patients.
Derk et al. [7]	A 57-year-old woman who was diagnosed SLE 16 years earlier and was diagnosed with sSS one year later.
Chevalier et al. [8]	A 41-year-old female who developed SLE 7 years after pSS.
Jodo et al. [9]	A 43-year-old female who had been diagnosed as pSS since 1986, then diagnosed SLE in 1990.
Zufferey et al. [10]	4 of 55 SS patients developed SLE (7.5%) after a mean follow-up of 12 years.
Manoussakis et al. [11]	In a cohort study of 283 SLE patients, SS preceded SLE in 18 of 26 SS-SLE.
Xu et al. [12]	A case control study of 41 SS-onset SLE and 214 SLE-only patients.
Yang et al. [13]	A case control study of 55 SS-onset SLE and 55 pSS patients.
Pan et al. [14]	In a group of 542 SLE patients, 17 of 35 SS-SLE showed pSS preceded SLE.
Taşdemir et al. [15]	A 16-year-old girl progressed SLE after 6 years diagnosis of SS.
Szanto et al. [16]	In a group of 56 SS-SLE patients, 8 presented SS preceding SLE by 1-18 years, and 22 presented SLE preceding SS by 1-19 years.
Satoh et al. [17]	A 69-year-old woman developed SLE after 10 years diagnosis of pSS.

SS, Sjögren's syndrome; pSS, primary Sjögren's syndrome; sSS, secondary Sjögren's syndrome.

Cystic lung disease (CLD) have been reported in several studies to occur in 7.4% to 46.2% of patients with SS [18–20, 22–24], and the finding of cyst-only disease appears rare [19, 20, 24, 41]. Only one study evaluated radiologic progression of CLD in SS, finding no progression in the size and number of cysts or pulmonary function decline in most individuals after a median 4-year follow-up [24]. The proof supporting a particular pathologic relevance of CLD in SS is insufficient. Two retrospective studies found that all individuals had cysts and nodules on imaging, one of those targeting 8 individuals with known lung amyloidosis and another showing 5 individuals suffering a known lymphoproliferative disorder [43, 44]. Besides, one retrospective study of 187 patients with biopsy-proven pulmonary amyloidosis showed that 11.2% presented CLD and a strong relevance with SS [45]. Another retrospective study evaluated 22 patients, 11 of which had SS, with LIP proven by biopsy: all patients had centrilobular nodules and ground-glass attenuation, seven had cysts [46]. And one study showed association of lymphoproliferative disorders, which defined as light-chain amyloidosis, monoclonal gammopathy, or malignant lymphoma with CLD [19], but this is not inconsistent with another study which found that CT patterns unlike nonspecific interstitial pneumonia (NSIP) rarely played a role in the prediction of a pathologic diagnosis [47].

Diffuse cystic lung disease (DCLD) is an uncommon clinical and radiographic presentation with a broad differential diagnosis [48]. Although diseases like LAM, pulmonary Langerhans cell histiocytosis and Birt-Hogg-Dubé syndrome continue to be the prototypical diffuse cystic pulmonary disorders most generally encountered in treatment centers or by pathologists [49, 50], cystic lung changes as the forme fruste of SS should also be concerned. Few studies have evaluated the characteristics of cystic abnormalities on chest radiographic testing in patients with SS [18, 19, 22, 25, 26]. It has been covered that the cystic pattern relating to SS has a typical manifestation on HRCT imaging. Characteristic features include a broad differentiation in cyst size and number, inner construction within cysts, perivascular and basilar predominant allocation, positional simplification of parenchymal structure generating a ‘dissolving lung appearance’ and frequent association with ground glass opacities and nodules [26]. Those discoveries can be characteristic enough to reduce the necessity for pulmonary biopsy even in defect of proved serologic results or salivary glands biopsy, especially in patients who cannot be tolerant of invasive diagnostic test.

We reported here a patient of pSS with diffuse cystic lung alterations developed SLE, no other reports have described both the SS-onset SLE and diffused cystic lung changes in one patient in details so far. The accurate cause of emerging SLE in patients with SS has not been well known yet. It is a pity that this patient did not perform laboratory and imaging test during 2014-2016 due to some private reasons so that we could not retrieve the dynamic profiles of laboratory parameters levels and imaging changes. While confirmatory invasive tests (bronchoalveolar lavage, transbronchial biopsy, surgical lung biopsy and so on) could not be carried out on this patient because of her difficulty in tolerating invasive procedure, we conjecture that lymphocytic interstitial pneumonia or NSIP is the most likely histopathology pattern upon imaging manifestations and beneficial response to prednisone therapy.

In summary, pSS with diffuse asymmetrical cystic lung changes can precede SLE by several years. When encounter patients of pSS with diffuse cystic lung manifestation emerging clinical and laboratory features of SLE, we should consider the possibility of emergence of SLE several years later. However, more clinical research is needed to establish definitive diagnosis criterion and effective treatment for those cases.
